# Retraction: Enhanced Translation of mRNAs Encoding Proteins Involved in mRNA Translation during Recovery from Heat Shock

**DOI:** 10.1371/journal.pone.0246558

**Published:** 2021-01-29

**Authors:** The PLOS ONE Editors

Following the publication of this article [1], concerns were raised regarding results presented in Figs 1, 2, 3, 4, and 5. Specifically,

- In Fig 1B, vertical discontinuities suggestive of image splicing were detected on either side of the 5 µL lane of the PABP1 panel.
- The PARP1 panel and the eEF1A2 panel in Fig 2A appear similar. The authors indicated that the wrong panel was used for the eEF1A2 results.
- The C (-RT) panel in Fig 2B appears to be devoid of any signal, including background signal usually expected in (negative) PCR results.
- In Fig 3A, vertical discontinuities suggestive of image splicing were detected between the first and second lane of both the PABP 1 C and PABP 1 R panels.
- In Fig 3A, the eEF1A1 HS panel appears similar to the eEF1A2 R panel when flipped, the eEF1A1 R panel appears similar to the eEF1A2 C panel when flipped, and the first and second lane of the eEF1A1 C panel appear similar to the second and third lane of the eEF1A2 HS panel when flipped. For the eEF1A1 C, eEF1A1R, eEF1A2 HS and eEF1A2 C gels, different quantification numbers are reported for bands that appear similar. The authors indicated that the wrong panels were used for the eEF1A2 results.
- In Fig 5A, vertical discontinuities suggestive of image splicing were detected between the second and third lane of the ZNF9 panel.
- The quantification results presented for the protein abundance in Figs 1C and 5C, the relative mRNA levels in Figs 2C and 4B, and the % of total mRNA results in Fig 3B do not always appear to match the blot and gel data presented in the related figures. In addition, there appears to be less variation in the results and the error bars than would be expected.
- The authors have provided photographs of lab book results underlying some of the published figures. However, the results provided did not include any markers or other control bands to confirm the results. In addition, some of the results presented cropped images of PCR and blot results only, as opposed to the full, uncropped figures underlying the published images.

In light of the concerns affecting multiple figure panels that question the integrity of these data and the data handling by the research group, the *PLOS ONE* Editors retract this article.

JB did not agree with the retraction and stands by the article’s findings. AKD either did not respond directly or could not be reached.
